# Privacy-Aware Early Detection of COVID-19 Through Adversarial Training

**DOI:** 10.1109/JBHI.2022.3230663

**Published:** 2022-12-20

**Authors:** Omid Rohanian, Samaneh Kouchaki, Andrew Soltan, Jenny Yang, Morteza Rohanian, Yang Yang, David Clifton

**Affiliations:** Department of Engineering ScienceUniversity of Oxford 6396 Oxford OX3 7DQ U.K.; Department of Engineering ScienceUniversity of Oxford 6396 Oxford OX3 7DQ U.K.; Oxford-China Centre for Advanced Research Suzhou 215123 China; Centre for Vision, Speech and Signal ProcessingUniversity of Surrey 3660 GU2 7XH Guildford U.K.; U.K. Dementia Research Institute Care Research and Technology CentreImperial College London 4615 SW7 2BX London U.K.; University of Surrey 3660 GU2 7XH Guildford U.K.; Queen Mary University of London 4617 E1 4NS London U.K.; Department of Engineering ScienceUniversity of Oxford 6396 Oxford OX3 7DQ U.K.; School of Public HealthShanghai Jiao Tong University 12474 Shanghai 200240 China; School of MedicineShanghai Jiao Tong University 12474 Shanghai 200240 China; John Radcliffe HospitalOxford University Hospitals NHS Foundation Trust 6397 OX3 7DQ Oxford U.K.; RDM Division of Cardiovascular MedicineUniversity of Oxford 6396 OX3 7DQ Oxford U.K.

**Keywords:** Adversarial machine learning, artificial neural networks, data privacy, deep learning, electronic medical records, medical information systems

## Abstract

Early detection of COVID-19 is an ongoing area of research that can help with triage, monitoring and general health assessment of potential patients and may reduce operational strain on hospitals that cope with the coronavirus pandemic. Different machine learning techniques have been used in the literature to detect potential cases of coronavirus using routine clinical data (blood tests, and vital signs measurements). Data breaches and information leakage when using these models can bring reputational damage and cause legal issues for hospitals. In spite of this, protecting healthcare models against leakage of potentially sensitive information is an understudied research area. In this study, two machine learning techniques that aim to predict a patient's COVID-19 status are examined. Using adversarial training, robust deep learning architectures are explored with the aim to protect attributes related to demographic information about the patients. The two models examined in this work are intended to preserve sensitive information against adversarial attacks and information leakage. In a series of experiments using datasets from the Oxford University Hospitals (OUH), Bedfordshire Hospitals NHS Foundation Trust (BH), University Hospitals Birmingham NHS Foundation Trust (UHB), and Portsmouth Hospitals University NHS Trust (PUH), two neural networks are trained and evaluated. These networks predict PCR test results using information from basic laboratory blood tests, and vital signs collected from a patient upon arrival to the hospital. The level of privacy each one of the models can provide is assessed and the efficacy and robustness of the proposed architectures are compared with a relevant baseline. One of the main contributions in this work is the particular focus on the development of effective COVID-19 detection models with built-in mechanisms in order to selectively protect sensitive attributes against adversarial attacks. The results on hold-out test set and external validation confirmed that there was no impact on the generalisibility of the model using adversarial learning.

## I. Introduction

Covid-19 has impacted millions across the world. Its early signs cannot be easily distinguished from other respiratory illnesses and hence an accurate and rapid testing approach is vital for its management. RT-PCR assay of nasopharyngeal swabs is a widely accepted gold-standard test, which has several limitations, including limited sensitivity and slow turnaround time (12–24 h in hospitals in high and middle-income countries). Several other techniques, including qualitative rapid-antigen tests (‘lateral flow’; LFTs), point-of-care PCR, and loop mediated isothermal amplification have been proposed [1], [2]. However, sensitivity results for for these techniques vary greatly amongst groups, with reported values ranging from 40% to 70%. [2], [3].

There are a number of research studies on the deployment of machine learning techniques to detect COVID-19 from various widely available features, including demographic and laboratory markers [4], [5]. A recent study introduced a machine learning test based on vital signs, routine laboratory blood tests and blood gas [6]. A strength of the test is the use of clinical data which is typically available within 1 h, much sooner than the typical turnaround time of RT-PCR testing. Current tests that employ machine learning are promising as they alleviate the need for specialised equipment, can potentially be more sensitive, and are faster than existing tests. Nonetheless they suffer from several shortcomings:

1) Most approaches that have appeared in the literature so far are based on basic machine learning techniques that require a complete retraining anytime a new batch of data is available. However, in a dynamic situation like a pandemic where new streams of data need to be processed, it is vital to incrementally learn from data without the need to start over and retrain the system using all the seen instances.
2) ML-based models explored in the COVID-19 literature are not equipped with an inherent mechanism to guard against possible issues that might arise due to the presence of demographic features. For example, models could easily get biased to a certain demographic group causing incorrect associations and overfitting.
3) Another issue is preserving the privacy of the patients and robustness against adversarial attacks. Most basic models can easily ‘leak’ information, making it easy for an adversary to recover sensitive information contained in the hidden representation. As blood tests are known to include features which typically correlate with demographic features, such as sex and ethnicity, exclusion of demographics does not necessarily solve the problem. For example, health issues like Benign Ethnic Neutropenia [7] or Sickle Cell Disease [8] are predominantly found in a certain number of ethnic groups and much less likely to occur in others. As an additional example, healthy men and women have different reference ranges for blood tests [9].

This work aims to address the above-mentioned shortcomings in existing research. The proposed adversarial architectures (Section IV) are designed to prevent the learning model from potentially encoding unwanted demographic biases and protect its sensitive information during the learning process. In the first architecture (Section IV-A), protection of attributes is explicit, with the option to select the attributes for guarding against adversarial attacks. Section V-C1 will investigate whether these direct protective measures would hurt generalisibility to unseen data.^1^^1^While the architecture introduced here is composed of simple fully connected layers, this adversarial setup can and has been tried in the literature with different architectures including logistic regression, SVMs, and LSTMS, among others. In the second architecture (Section IV-B), protecting attributes is based on a general adversarial regularisation and is not tied to any specific subset of selected attributes.

The proposed models in this study are designed to preserve sensitive information against adversarial attacks, allow incremental learning, and reduce the potential impact of demographic bias. However, the main focus of the work is in privacy preservation. The contributions of this work are as follows:

• Two adversarial learning models are introduced for the task of COVID-19 identification based on Electronic health records (EHR). The models perform satisfactorily on a real COVID-19 dataset and in comparison with strong baselines. Unlike conventional tree-based methods, these architectures are well-suited for transfer learning, multi-modal data, and other advantages of neural models without a significant performance trade-off.
• The models use adversarial regularisation to make them robust against leakage of sensitive information and adversarial attacks and suitable for scenarios where preservation of privacy is important or classification bias is costly.
• A series of tests are performed to quantitatively demonstrate the efficacy of the proposed architectures in protecting sensitive information against adversarial attacks in comparison with a neural model that is not adversarially trained.
• Several experiments are done in order to observe the effect of this type of training on generalisability across different demographic groups.
• The models are externally validated using data from other hospital groups.

## II. Privacy Attacks in Machine Learning and Healthcare

**Fig. 1.**
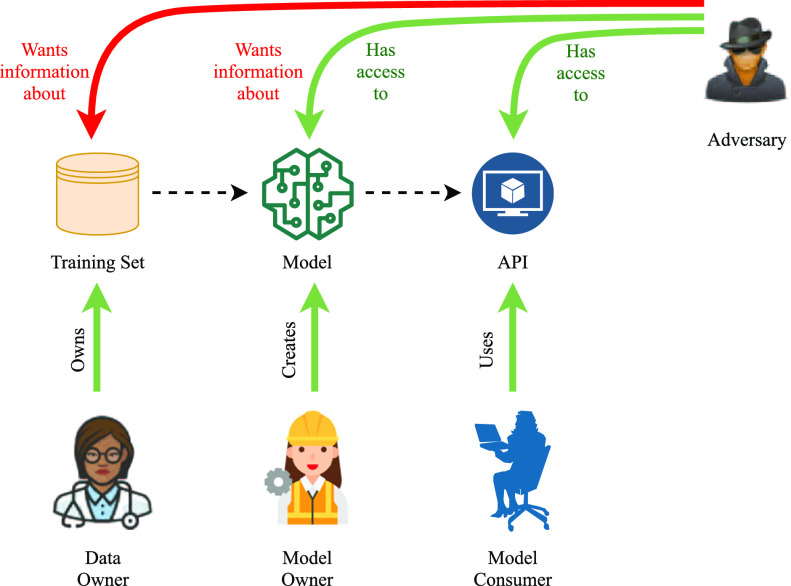
Schematic view of privacy attacks for a machine learning model. Dashed lines represent information flow, and full lines signify possible actions.

There are various ways a trained model can be attacked by an adversary. The goal in most of them is to infer some kind of knowledge that is not originally meant to be shared or is unintentionally encoded by the model. At least three different forms of attack are known, namely, membership inference, property inference, and model inversion [10]. In this work, the focus is on property inference, in which an adversary who has access to model's parameters during training, tries to extract information about certain properties of the training data that are not necessarily related to the main task. Fig. 1 shows the general overview of privacy attacks according to Rigaki and Garcia [11]. The adversary, in the particular setup in this study, can see the model and its parameters and wants information about the data to which they do not have direct access.

Attacks of this kind are possible in any scenario where the model is stored and trained on an external server. Protecting an ML model against property inference attacks is especially useful in the context of collaborative and federated learning, where models locally train on different portions of the dataset and share their parameters over a network that might or might not be fully secure against eavesdropping [12].

Within the context of healthcare, such attacks can reveal sensitive personal data and prove disastrous for hospitals. GDPR defines personal data as ‘any information relating to an identified or identifiable natural person’. Article 9(1) of the GDPR declares the following types of personal data as sensitive: data revealing racial or ethnic origin, political opinions, religious or philosophical beliefs, or trade union membership, genetic and biometric data, and data concerning health or sex life or sexual orientation of the subject [13].

Sensitive information such as age, gender, location, or ethnicity are usually quantised or anonymised in large healthcare datasets. However, as demonstrated in Section V-C, this information can be easily recovered by a simple attack model because of the implicit associations that exist between such information and other features in the dataset.

Property inference attacks are not limited to recovering any specific type of data and can predict both categorical and numerical values. For instance, they can be used to train attacker models that learn to identify both demographic features (implicitly present in the data) and blood test features (explicitly present) that highly correlate with certain diseases. It is then possible to use this trained model to re-identify some patients based on their demographic features and possible combination of diseases [14].

## III. Task Definition

In the binary classification setting, each neural network $f$ is trained to predict labels $y_{1}$, $y_{2}$,..., $y_{n}$ from instances $x_{1}$, $x_{2}$,..., $x_{n}$. Each instance $x_{i}$ contains a set of sensitive (in this case demographic) discrete features $z_{i} \in {1,2,{\ldots },k}$ which should be “protected”.^2^^2^Ideally the transformation $y_{i} = f(x_{i})$ should not to be confounded by specific values of $z_{i}$. However, the experiments here are focused on privacy preservation and not on the closely related subject of debiasing. These sensitive features are called *protected attributes*.

In the context of classification, any neural network $f(x)$ can be characterised as an encoder, followed by a linear layer $W: f(x) = W \times h(x)$. $W$ can be seen as the last layer of the network (i.e. dense + softmax) and $h$ is all the preceding layers [15].

Suppose there exists an *attacker* model $f_{att}$ that is trained on the encoder $h(x)$ of a neural classifier in order to predict $z_{i}$. If this trained adversary is able to predict $z_{i}$ based on the encoded representation from the model, the model has *leaked* and privacy of the model has been compromised.

It is unlikely that $h(x)$ would be completely guarded against an attack. If it encodes sufficient information about $x_{i}$, it might reveal some information to a properly trained $f_{att}$. The trained model $f$ is said to be private with regards to $z_{i}$ if an attacker model $f_{att}$ that has access to $f$’s encoder ($h(x)$) cannot predict $z_{i}$ with a greater probability than a majority class baseline.

If $h(x)$ is perturbed too much, it will not be informative to $f_{att}$ but would also fail in accurately predicting the main task label $y_{i}$. Therefore, the challenge is to ensure privacy against potential attackers with regards to the protected attributes while achieving a reasonably good result in the main task.

## IV. Methodology

In this work, a standard supervised learning scenario is followed where each training instance $x_{i}$ represents information from blood tests and vital signs for each patient seen at the hospital and $y_{i}$ is the corresponding Boolean value denoting the result of the PCR test for that patient. The task is to train a model to predict the correct label for each patient.

### A. Adversarial Training Based on Gradient Reversal

**Fig. 2.**
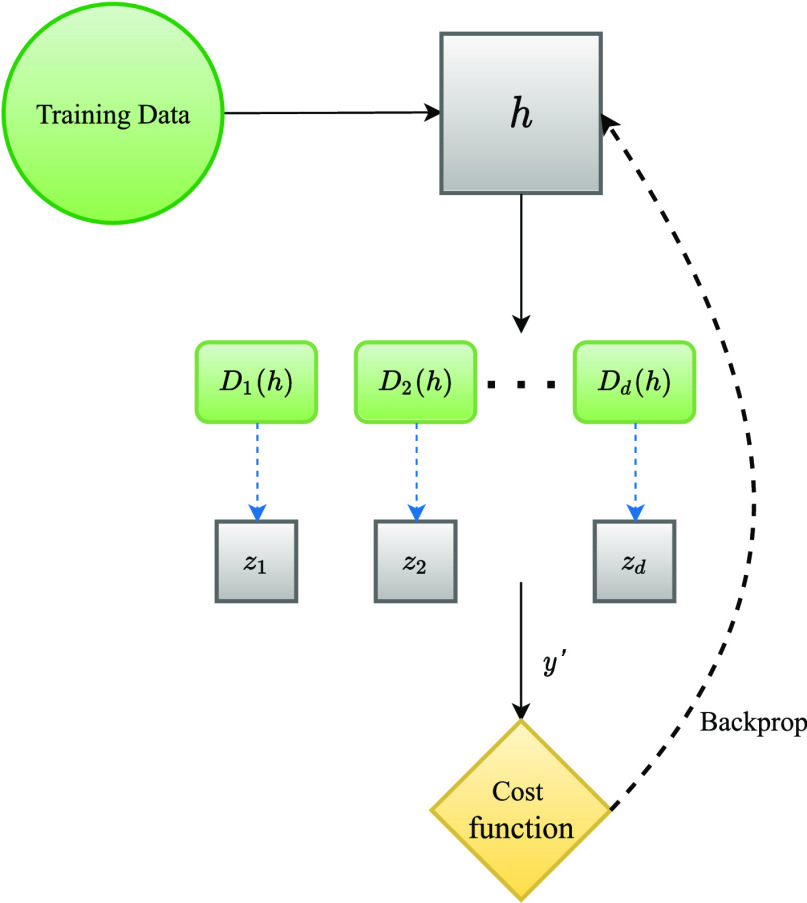
Overall structure of the proposed model. Each $D_{i}$ is a discriminator that aims to predict any of the $d$ categorical features $z_{i}$.

The first adversarial architecture that is explored in Fig. 2 is comprised of one main part and a number of secondary networks:

1) A main classifier $M$ that is the central component of the model. It consists of a stack of $n$ fully connected layers with dropout and batch normalisation, followed by a softmax layer at the end.
2) $d$ networks with auxiliary objectives separate from the main task. Given $d$ categorical features, each of these secondary networks (henceforth referred to as discriminators) predict the value for that feature given each training instance.

Assume $h_{i}$ is the representation of an instance at the *i*th layer within $M$. This is the point of interception where the auxiliary networks get access to the contents of $M$. All these components then train in tandem with the following loss function:

\begin{equation*}
L = L_{M} - \sum _{i=1}^{d}\lambda L_{D_{i}} \tag{1}
\end{equation*}

Each $D_{i}$ corresponds to a separate discriminator network that predicts one of the $d$ different categorical features of interest. $\lambda$ is a weighting factor and can control the contribution of each individual auxiliary loss. Formula 1 is set up so that after backpropagation, the contents of $h$ be maximally informative for the main task, and minimally informative for prediction of the protected features. Loss of the main task is computed using binary cross entropy.

If $x$ and $y$ are the features and labels, $\hat{y}$ and $\hat{z}$ the predictions for the main target and protected features, $\theta _{M}$ and $\theta _{D_{i}}$ the parameters of the main classifier and its $d$ discriminators, and $L$ is the joint binary cross entropy loss function, the training objective can be formulated as finding the optimal parameters $\hat{\theta }$ such that:

\begin{align*}
\hat{\theta } =& \min_{\theta _{M}} \max_{\left\lbrace \theta _{D_{i}} \right\rbrace _{i=1}^{d}} L \left(\hat{y}\left(x;\theta _{M}\right),y\right) \\
& - \lambda \sum _{i=1}^{d} L \left(\hat{z}\left(x;\theta _{D_{i}}\right),z_{i}\right)\tag{2}
\end{align*}

#### 1) Gradient Reversal Layer

As discussed in Section IV-A, during training, the objective is to jointly minimise both of the following terms:^3^^3^The formulation of GRL in this section is based on [16].

\begin{align*}
& arg \ \min _{D} L\left(D\left(h(x_{i})\right),z\right) \tag{3}\\
& arg \ \min _{h, c} L\left(c\left(h(x_{i})\right),y_{i}\right) - L\left(D\left(h(x_{i})\right),z\right) \tag{4}
\end{align*}

where each $x_{i}$ is an instance of the data which is associated with the protected attribute $z$. $D$ is the discriminator (the adversarial network), and $c$ is the classifier used to predict the labels for the main task from representation $h$. $L$ denotes the loss function.

Using an optimisation trick called the *Gradient Reversal Layer (GRL)*, the above terms can be combined into a single objective. This idea was first introduced in the context of domain adaptation [17] and was later also applied to text processing [16], [18]. GRL is easy to implement and requires adding a new layer to the end of the Discriminator's encoder.

During forward propagation, GRL acts as an identity layer, passing along the input from the previous layer without any changes. However, during backpropagation, it multiplies the computed gradients by $-1$. Mathematically this layer can be formulated as a pseudofunction with the following two incompatible equations:

\begin{equation*}
{\begin{cases}GRL(x) = x & \text{if in forward mode}\\
\frac{dGRL(x)}{dx} = - I & \text{if in backprop mode} \end{cases}} \tag{5}
\end{equation*}

Using this layer, the loss function can be consolidated into one single formula, and a single backpropagation would suffice in each training epoch. For the trivial case of having only one protected attribute, equations 3 and 4 can be combined as follows:

\begin{equation*}
arg \ \min _{h,c,D} L\left(c\left(h(x_{i})\right),y_{i}\right)+L\left(D\left(\lambda GRL\left(h(x_{i})\right)\right),z\right) \tag{6}
\end{equation*}

The objective is to minimise the total loss, and for the case of the discriminator, the gradients are reversed and scaled by $\lambda$. It is straightforward to generalise this to the case where there are multiple (in the case of this study 3; namely, age, gender, and ethnicity) protected attributes and corresponding $D_{i}$s:

\begin{equation*}
L = L_{M} + \sum _{i=1}^{d} L(D_{i}(\lambda GRL(h(x),z_{i}))))) \tag{7}
\end{equation*}

### B. Adversarial Training Based on Fast Gradient Sign Method

As the second adversarial architecture, another model is developed in which the adversarial component can perturb the representation during training with some added noise. The direction of this noise (i.e. whether the added noise is a positive or negative number) is dependent on the signs of the computed gradients.

This adversarial method is based on linear perturbation of inputs fed to a classifier. In every dataset, the measurements enjoy a certain degree of precision, below which could be considered negligible error $\epsilon$. If $x$ is the representation of an instance, it is likely that the classifier would treat $x$ the same as $\tilde{x}=x+\eta$, as long as $\Vert \eta \Vert _{\infty } < \epsilon$.

However, this small perturbation grows when it is multiplied by a weight matrix $w$:

\begin{equation*}
w^{\top }\tilde{x} = w^{\top }(x+\eta) = w^{\top }x+w^{\top }\eta \tag{8}
\end{equation*}

The perturbation is maximised when $\eta = sign(w)$, predicated on the assumption that it remains within the max-norm constraint defined above. In the context of deep learning, the method can be formulated in the following way:

If $\theta$ is the parameters of the model, and $J$ is the cost function, during training, for each instance a perturbation of $\eta$ is added to the representation of the instance such that:

\begin{equation*}
\eta = \epsilon sign\left(\triangledown _{x} J\left(\theta,x,y_{pred}\right)\right) \tag{9}
\end{equation*}

This procedure is known as the *fast gradient sign method (FGSM)*, originally introduced in a seminal 2015 paper by Goodfellow et al. [19]. It can be viewed either as a regularisation technique or a data augmentation method that includes unlikely instances in the dataset. For training, the following adversarial objective function can be used:

\begin{align*}
&\tilde{J}\left(\theta,x,y_{pred}\right) = \alpha J(\theta,x,y) \\
&\qquad + (1-\alpha)J\left(\theta,x + \epsilon sign\left(\triangledown _{x} J\left(\theta,x,y_{pred}\right)\right)\right) \tag{10}
\end{align*}

This method can be seen in terms of making the model robust against worst case errors when the data is perturbed by an adversary [19]. Because of this regularisation, the expectation is that hidden representations would become less informative to an attacker network that attempts to retrieve demographic attributes. Following the original paper, $\alpha$ is usually taken to be 0.5, which turns the equation into a linear combination with equal weights given to both terms in the objective function.

**Fig. 3.**
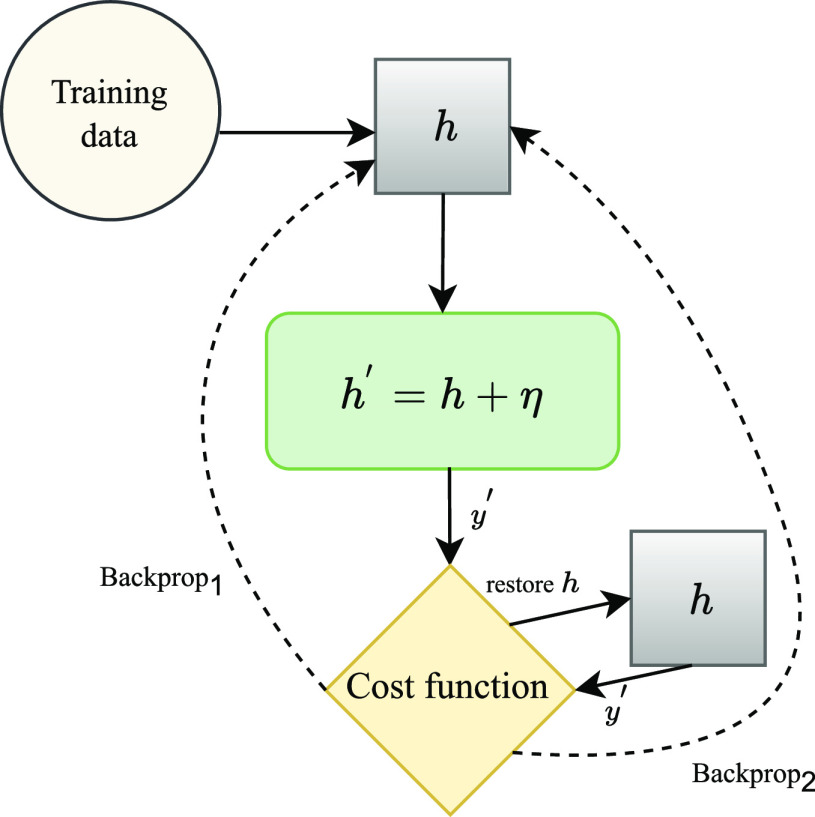
Overall structure of FSGM. $y^{\prime}$ is the predicted label. $\eta$ is added noise at the point of interception $h$.

In the implementation used for this work (Fig. 3), alongside the main component, there is an attacker that intercepts the model at a certain step during each training epoch, makes a copy of the pre-attack parameters in the intercepted layer, and injects noise into the model. Based on this information, an adversarial loss is computed and backpropagation is applied.

After this step, a restore function is executed, returning the parameters of the intercepted layer back to its pre-attack values. A regular loss is then computed and backpropagation is applied for a second time. This added noise is computed based on (9). If $h$ is the representation of a training instance at the time of interception by the attacker, the perturbation is calculated by $h^{\prime} = h+ \eta$.

### C. Dataset

A hospital dataset referred to as OUH is used for the studies in this study. OUH is a de-identified EHR dataset, covering unscheduled emergency presentations to emergency and acute medical services at Oxford University Hospitals NHS Foundation Trust (Oxford, U.K.). These hospitals consist of four teaching hospitals, which serve a population of 600,000 and provide tertiary referral services to the surrounding region. At the time of model development, linked deidentified demographic and clinical data were obtained for the period of November 30, 2017 to March 6, 2021. For each presentation, data extracted included presentation blood tests, blood gas results, vital sign measurements, results of RT-PCR assays for SARS-CoV-2, and PCR for influenza and other respiratory viruses. Patients who opted out of EHR research, did not receive laboratory blood tests, or were younger than 18 years of age have been excluded from this dataset.

For OUH, hospital presentations before December 1, 2019, and thus before the global outbreak, were included in the COVID-19-negative cohort. Patients presenting to hospital between December 1, 2019, and March 6, 2021, with PCR confirmed SARS-CoV-2 infection, were included in the COVID-19-positive cohort. This period includes both the first and second waves of the pandemic in England.^4^^4^[Online]. Available: https://coronavirus.data.gov.uk/details/cases Because of incomplete penetrance of testing during early stages of the pandemic and limited sensitivity of PCR swab tests (around 70%), there is uncertainty in the viral status of patients presenting during the pandemic who were untested or tested negative. Therefore, these patients were excluded from the datasets.

**Fig. 4.**
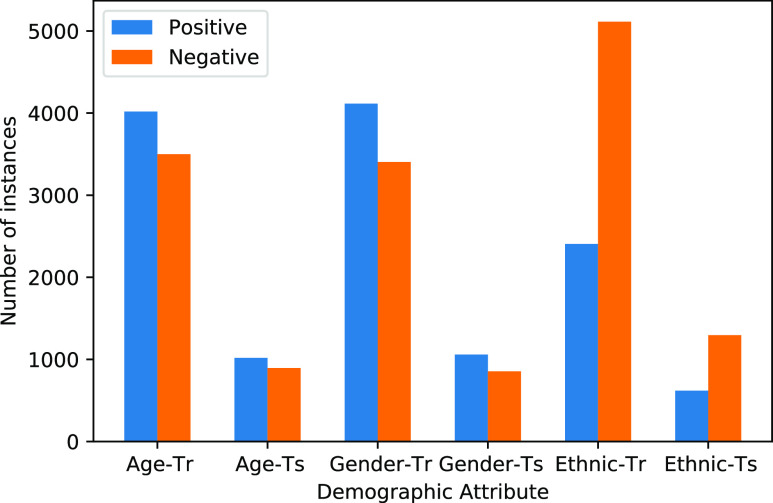
Distribution of labels for each demographic attribute in TRAIN(-Tr) and TEST(-Ts) sets in OUH.

There are 3081 instances of COVID-19-positive in the original dataset and 112121 negative instances. For the experiments with OUH, the majority class was subsampled in order to reach a more balanced dataset with prevalence 0.5 (i.e. 6162 total labels). Age, gender, and ethnicity information were binarised during preprocessing. For gender, the average age is 64, which is taken as cut-off point for binarisation.^5^^5^As there was not a big gap between median and mean, the mean was used. The ethnicity information, which were encoded using NHS ethnic categories, were divided into white and non-white. No significant difference in numbers or general patterns was observed in the case where ambiguous ethnic categories were removed as opposed to labelling them as non-white. Therefore only the latter is reported. While quantising features in this way involves oversimplification and loss of detail, it keeps the values binary across all the protected attributes making comparisons easier in the experimental setup. This setup is designed to make it easy for the attacker (i.e. having to decide between 5 different age brackets as opposed to two would be a much harder problem to solve for the attacker). If the training setup is effective in the binary case, it is more likely to be effective in a multi-class scenario. Fig. 4 shows the distribution of demographic labels in the OUH dataset.

In Section V-C2, the proposed models will be externally validated on three NHS Foundation Trust datasets [20], namely Bedfordshire Hospitals NHS Foundation Trust (BH), University Hospitals Birmingham NHS Foundation Trust (UHB), and Portsmouth University Hospitals NHS Trust (PUH). The entire test sets in their original label distribution within the pandemic time-frame are used to make sure the evaluation is fair and that it mirrors the highly imbalanced data used in hospitals. Table I shows the statistics for the Covid-19 Positive cases in the datasets.

**TABLE I:** Label Distributions for PCR (Along With Percentage of Each Label) for UHB, BH, and PUH Datasets Used for External Validation of the Models

	COVID+	COVID-	total
UHB	624 (1.48%)	42095 (98.52%)	42719
BH	209 (11.13%)	1669 (88.87%)	1878
PUH	2002 (5.2%)	36579 (94.8%)	38581

Evaluation at UHB trust considered all patients presenting to The Queen Elizabeth Hospital, Birmingham, between December 01, 2019 and October 29, 2020. The Queen Elizabeth Hospital is a large tertiary referral unit within the UHB group which provides healthcare services for a population of 2.2 million across the West Midlands. Confirmatory COVID-19 testing was performed by laboratory SARS-CoV-2 RT-PCR assay.

Evaluation at BH considered all patients presenting to Bedford Hospital between January 1, 2021 and March 31, 2021. BH provides healthcare services for a population of around 620,000 in Bedfordshire. Confirmatory COVID-19 testing was performed by point-of-care PCR based nucleic acid testing [SAMBA-II & Panther Fusion System, Diagnostics in the Real World, U.K., and Hologic, USA].

Evaluation at PUH considered all patients admitted to the Queen Alexandria Hospital, serving a population of 675,000 and offering tertiary referral services to the surrounding region, between March 1, 2020 and February 28, 2021. Confirmatory COVID-19 testing was by laboratory SARS-CoV- 2 RT-PCR assay.

Validation cohorts are prepared based on the works presented by Soltan et al. in [20] and [6]. Held-out test sets are generated by an 80:20 split stratified by patients with COVID-19 and balanced equally with pre-pandemic controls.

## V. Experiments and Results

A series of experiments are performed in order to test the proposed models and compare them against baselines. The baseline non-adversarial model which is used as the basic structure to start from, consists of 3 fully connected dense layers with batch normalisation and dropout. This model is referred to as Base. During 10-fold cross-validation, the best hyperparameters were chosen using random search. It was empirically found that heavy hyperparameter optimisation had at best mixed results and adding more layers to the model did not consistently boost performance. For this reason, a set of parameters that seemed to work well across all the models during cross-validation were kept fixed (Table II).^6^^6^All the models with the exception of ADV_per_ were trained for 15 epochs for experiments on OUH. For external validation, this was set to 30 epochs. ADV_per_ seemed to require more training epochs in all the experiments, therefore it was trained with 30 epochs for both OUH and external validation. The Base model was also kept simple with only a few layers in order to have direct and straightforward comparisons with the adversarially trained models. The demographic-based adversarial model is referred to as ADV and its main component is the same as Base. Since after training, only the Base part will be tested (i.e. discriminators will detach), the ADV model ends up having the exact same number of parameters as Base. The perturbation-based adversarial model, which also has the same number of parameters as Base, is referred to as Adv_per_. All the reported results on the test set are the median of three consecutive runs. The experiments are performed using an Intel Xeon W-2223 CPU (3.60 GHz) processor equipped with a Quadro P400 GPU.

**TABLE II:** Hyperparameter Values Used for All the Experiments

learning rate	$\lambda$	batch size	hidden dimension (Base)	hidden dimension (disc)	dropout	epochs
0.0008	2	16	150	300	0.5	15/30

In what follows, the feature sets used and the train and test procedure are explained. Finally, the main task and attacker results are reported under different scenarios.

### A. Feature Sets

Two sets of clinical variables were investigated (Table III): presentation blood tests from the first blood draw on arrival to hospital and vital signs. Only blood test markers that are commonly taken within existing care pathways and are usually available within 1 h in middle and high-income countries were considered here.

**TABLE III:** Clinical Parameters Included in Each Feature Set

Feature Type	Features included
Presentation blood tests	Haemoglobin, haematocrit, mean cell volume, white cell count, neutrophil count, lymphocyte count, monocyte count, eosinophil count, basophil count, platelets, prothrombin time, INR, APTT, sodium, potassium, creatinine, urea, eGFR, C Reactive Protein (CRP), albumin, alkaline phosphatase, ALT, bilirubin
Presentation vital signs	Heart rate, respiratory rate, oxygen saturation, systolic blood pressure, diastolic blood pressure, temperature, oxygen flow rate

### B. Training and Testing

The models are trained and tested in a binary classification task in which the labels are confirmed PCR test results. As the first step, the model is evaluated on the TRAIN set in a stratified 10-fold cross-validation scenario during which a threshold is set on the ROC curve to meet the minimum recall constraint.^7^^7^The idea behind calibration of recall is to make sure the false negatives do not exceed beyond a certain point. In a hospital setting and in a pandemic, it is too costly to send patients home with a false negative result or transfer them to wards and potentially expose other inpatients to infection. Therefore, high sensitivity is needed to give physicians confidence that negative results are truly negative. Consequently, the model is trained on the TRAIN set and tested on the holdout TEST data and results are computed using the previously set threshold.

**Fig. 5.**
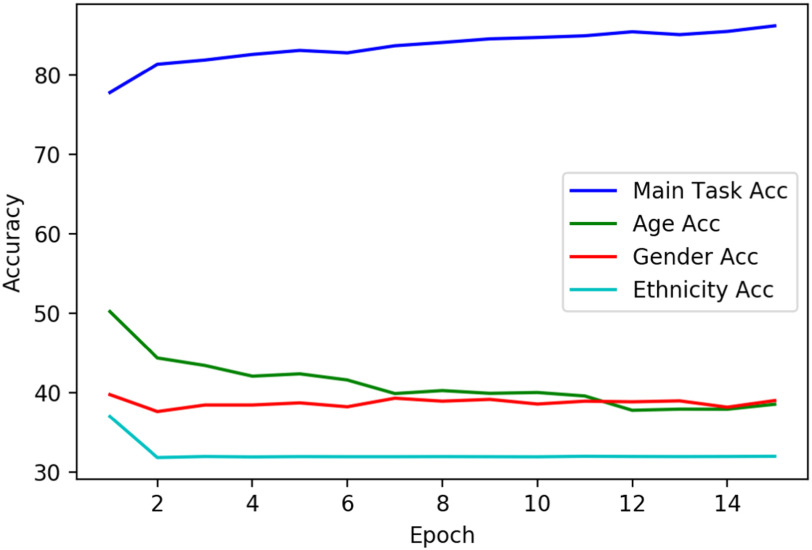
Accuracy scores for the main and each of the three discriminators for each epoch.

During training of the ADV model, the expectation is that the accuracy of the main classifier increase over subsequent epochs, and since the learning setup is such that discriminators are constantly misled, performance is intended to be kept below or around 50% accuracy. To test this assumption, the changes in the trajectory of accuracy for the main and three auxiliary tasks are plotted in the first 15 epochs. This is when the ADV model is being trained on TRAIN set and before it is tested on holdout TEST. As can be seen in Fig. 5, accuracy for the main task keeps growing steadily while discriminator accuracy drops below 50% and plateaus afterwards.

In Table IV the results are reported on the main task of predicting PCR results for all the models. The results demonstrate that the models perform well at the main task, namely, predicting the outcome of the PCR test.

**TABLE IV:** Results for the 4 Different Models for the OUH Dataset in a 10-Fold Cross-Validation Setting With the Recall Value Set as $0.8\pm 0.07$

Model	Recall	Precision	F1-Score	Accuracy	Specificity	PPV	NPV	AUC	Threshold
Base	0.7335	0.7356	0.7341	0.8219	0.8670	0.7356	0.8667	0.8623	0.1551
ADV_per_	0.7331	0.7355	0.7338	0.8216	0.8668	0.7355	0.8665	0.8571	0.0464
ADV	0.7355	0.7308	0.7325	0.8199	0.8629	0.7308	0.8670	0.8553	0.1669

### C. Attacking Trained Networks to Predict Protected Attributes

In order to asses how much privacy each model can provide against an adversarial attack, a series of experiments are performed in which 3 different non-adversarial Base models are trained on the training data, with each corresponding to the prediction of a different demographic attribute. In other words, instead of predicting the PCR test result, a protected attribute is provided as the label to train and test on. The experiments are performed under the same conditions as the main task. The attacker is first trained in a 10-fold cross-validation scenario and a threshold is set based on the ROC curve with the minimum recall constraint of $0.8\pm 0.07$.

Subsequently, the attackers are trained on TRAIN set and tested on the TEST portion of the dataset and predict the same values given the obtained threshold set during 10-fold CV. These results are important to the final interpretations of the model privacy because they determine the upper bound for the most amount of leak the proposed models can have. In Table V, the results are reported for trained attackers on the TEST portion of the dataset given each protected attribute that was predicted.

**TABLE V:** Attacker Results on the TEST Set When Trained and Tested on Features Directly. This Serves as the Upper Bound for Information Leakage

Predicted Attribute	Recall	Precision	F1-Score	Accuracy	Specificity	PPV	NPV	AUC
Age	0.7193	0.7470	0.7329	0.7936	0.8782	0.74704	0.8622	0.8884
Gender	0.7346	0.78897	0.7608	0.8092	0.9017	0.7889	0.8717	0.9104
Ethnicity	0.6688	0.4506	0.5384	0.6170	0.5922	0.4506	0.7815	0.6737

The lower bound is the the majority class baselines in which the attacker simply relies on some prior information about the distribution of the protected attributes to predict these features and does not make use of the obtained hidden representations. For instance, if a dataset is obtained in Scotland, relying on the known fact that the predominant ethnic category is British White, the attacker would simply assign the same label to all of the instances. Statistics about majority classes for each attribute is given in Table VI in both TRAIN and TEST sets. As can be seen, ethnicity is the most unbalanced category in comparison with gender and age in which class labels are more equally distributed.

**TABLE VI:** Percentage of Majority Class Labels to the Whole Data for Each Demographic Attribute

Protected attribute	TRAIN	TEST
Age	0.53	0.53
Gender	0.54	0.55
Ethnicity	0.68	0.67

As the next step, the baseline and proposed adversarial models are trained on the TRAIN set and the weights of the neural networks are saved. Subsequently, the trained attackers are loaded and tested, not on the feature directly this time, but on the output of the encoder of the baseline and adversarially trained models. The idea is that, if an adversarially trained model is indeed protecting demographic attributes, it should make it harder for an attacker to predict those values from its encoded representations in comparison with a baseline model that is not specifically designed for preservation of privacy. Results shown in Table VII already show a degree of privacy provided by the non-adversarial encoder, as they indicate a noticeable decrease in performance compared to Table V. The most marked decrease is visible in prediction of gender, in which performance drops from AUC of 0.9104 to 0.6926. In the case of age, however, the attacker seems more robust.

**TABLE VII:** Attacker Results on the TEST Set When Trained and Tested on the Output Generated by the Encoder of the NonAdversarial Base Model

Predicted Attribute	Recall	Precision	F1-Score	Accuracy	Specificity	PPV	NPV	AUC
Age	0.7831	0.5855	0.6701	0.7549	0.7229	0.5855	0.8695	0.8131
Gender	0.7969	0.4336	0.5616	0.6551	0.4795	0.4336	0.8252	0.6926
Ethnicity	0.7835	0.3776	0.5096	0.4932	0.3544	0.3776	0.7660	0.6265

Since the intention is to keep the attackers blind to the encoding strategy used by the adversarially trained model, in order to test the attackers on the ADV and ADV_per_ models, the same threshold which was set during 10-fold CV on the encoded representation of the Base model is used here as well. Therefore, to predict the three attributes, the attacker which is trained on the non-adversarial encoder on the TRAIN set is loaded and then tested on the ADV/ADV_per_ model's encoder.

The results in Tables VIII and IX confirm the assumption that an adversarial learning procedure, either with separate discriminator networks for each protected attribute or using perturbation-based regularisation, provides a greater level of privacy against attacks by an intruder that intends to recover this information using a representation obtained from the model.

**TABLE VIII:** Attacker Results on the TEST Set When Trained on the Encoder of the Base Model and Tested on the Encoder of the ADV Model

Predicted Attribute	Recall	Precision	F1-Score	Accuracy	Specificity	PPV	NPV	AUC
Age	0.8213	0.3439	0.4849	0.5386	0.2167	0.3439	0.7082	0.5744
Gender	0.6676	0.3117	0.4250	0.4869	0.2631	0.3117	0.6129	0.4572
Ethnicity	0.4394	0.3493	0.3892	0.5417	0.5907	0.3493	0.6782	0.5112

**TABLE IX:** Attacker Results on the TEST Set When Trained on the Encoder of the Base Model and Tested on the Encoder of the ADV_per_ Model

Predicted Attribute	Recall	Precision	F1-Score	Accuracy	Specificity	PPV	NPV	AUC
Age	0.4946	0.3164	0.3859	0.4811	0.4659	0.3164	0.64835	0.4723
Gender	0.6421	0.3269	0.4333	0.5067	0.3391	0.3269	0.6546	0.5189
Ethnicity	0.4151	0.3395	0.37355	0.5376	0.5961	0.3395	0.6709	0.4870

#### 1) Demographic Cross-Testing to Asses Generalisability

The application of an adversarial learning procedure to protect selected attributes involves a training setup with competing losses which is intended to weaken undesirable implicit associations contained in the hidden representations of the network. This is expected to result in a certain amount of performance drop compared to the non-adversarial baseline. As long as this drop is not massive, the performance-privacy trade-off is justified. However, a more general concern is whether a model like ADV, with its 3 different discriminators and the direct and specific manipulation of its hidden representations would generalise poorly when tested on certain demographic sub-populations of the dataset. Since ADV_per_ applies its regularisation without specifically targeting any protected attributes, it is less likely to suffer from this issue.

In order to investigate whether protecting demographic attributes damages generalisability of the ADV, a series of experiments were performed with the aim to train the Base and ADV models only on one demographic group and then test them on the other group. The adversarial model is compared with the baseline to make sure that generalisability of the ADV model is not hurt. Since there are 3 different binary attributes, there are 6 possible ways to cross-test the models. These subgroups are denoted with f (female), m (male), w (white), n (non-white), o (old), and y (young).^8^^8^Old and young here are simply labels to distinguish the two age sub-groups and do not necessarily reflect notions of young and old in society. To restructure the dataset for these experiments, in each case all the data is combined and TRAIN and TEST are filtered based on the targeted demographic. For example ‘m2f’ would mean that the TRAIN set only contains females and the TEST set only males. The results in Table X clearly indicate that adversarial learning has not damaged generalisability in any of scenarios in which the Base and ADV models were tested.

**TABLE X:** Results of Demographic Cross-Tests to Assess the Effects of Adversarial Training on Generalisability Across Different Subgroups of the Dataset

Cross-test	Model	Recall	Precision	F1-Score	Accuracy	Specificity	PPV	NPV	AUC
f2m	Base	0.6876	0.7401	0.7100	0.8119	0.8768	0.7401	0.8501	0.8435
f2m	ADV	0.7130	0.7310	0.7191	0.8137	0.8663	0.7310	0.8591	0.8452
m2f	Base	0.6720	0.7520	0.7085	0.8145	0.8889	0.7520	0.8449	0.8403
m2f	ADV	0.6947	0.7405	0.7142	0.8137	0.8759	0.7405	0.8527	0.8389
n2w	Base	0.6771	0.7428	0.7057	0.8115	0.8813	0.7428	0.8466	0.8406
n2w	ADV	0.6971	0.7281	0.7105	0.8101	0.8687	0.7281	0.8524	0.8397
w2n	Base	0.6802	0.7442	0.7077	0.8126	0.8815	0.7442	0.8479	0.8424
w2n	ADV	0.7037	0.7302	0.7149	0.8122	0.8686	0.7302	0.8552	0.8428
o2y	Base	0.6873	0.7449	0.7123	0.8140	0.8800	0.7449	0.8502	0.8449
o2y	ADV	0.7019	0.7344	0.7153	0.8131	0.8709	0.7344	0.8550	0.8435
y2o	Base	0.6716	0.7425	0.7021	0.8098	0.8817	0.7425	0.8445	0.8379
y2o	ADV	0.6922	0.7238	0.7055	0.8066	0.8660	0.7238	0.8501	0.8364

#### 2) External Validation of the Models

In order to validate the models on external data, Base, ADV, and ADV_per_ are trained on the OUH dataset (as described in Section IV-C) and later tested on the entirety of the UHB, BH, and PUH datasets. The same procedure is followed as the previous experiments: First 10-fold CV is run on the OUH dataset and a threshold is set, and then the models are tested on the external test data with the previously obtained threshold. The hyperparameters were kept the same for these experiments with the exception of ADV_per_ which seemed to converge better after 30 epochs during 10-fold CV. Tables XI, XII, and XIII show the results of this experiment on the UHB, BH, and PUH test sets, respectively.

**TABLE XI:** Results for the Models When Trained on OUH and Tested on the UHB Dataset

Model	Recall	Precision	F1-Score	Accuracy	Specificity	PPV	NPV	AUC
Base	0.7371	0.7261	0.7316	0.8602	0.8609	0.7261	0.8675	0.8643
ADV_per_	0.7155	0.7286	0.7218	0.8657	0.86669	0.7286	0.8591	0.8531
ADV	0.7275	0.7236	0.7256	0.8602	0.8611	0.7236	0.8634	0.8586

**TABLE XII:** Results for the Models When Trained on OUH and Tested on the BH Dataset

Model	Recall	Precision	F1-Score	Accuracy	Specificity	PPV	NPV	AUC
Base	0.6556	0.7760	0.7045	0.8795	0.9002	0.7760	0.8414	0.8608
ADV_per_	0.6301	0.7562	0.6767	0.8746	0.8933	0.7562	0.8320	0.8115
ADV	0.6923	0.7473	0.7163	0.8690	0.8806	0.7473	0.8521	0.8506

**TABLE XIII:** Results for the Models When Trained on OUH and Tested on the PUH Dataset

Model	Recall	Precision	F1-Score	Accuracy	Specificity	PPV	NPV	AUC
Base	0.6988	0.7441	0.7168	0.8638	0.8762	0.7441	0.8545	0.8567
ADV_per_	0.6401	0.7575	0.6858	0.8768	0.8937	0.7575	0.8351	0.8173
ADV	0.6973	0.7450	0.7184	0.8680	0.8788	0.7450	0.8537	0.8527

## VI. Discussion and Conclusion

In this work, two adversarially trained models were introduced and evaluated for the task of predicting COVID-19 PCR test results based on routinely collected blood tests and vital signs. The data was processed in the form of tabular data.

In the experiments, the focus was on the issue of leakage of potentially sensitive attributes that are implicitly contained in the datasets, and how an attacker network can successfully retrieve this information under different circumstances. Information like age seemed to be easily inferred with high accuracy from the features or from the hidden representation of the Base model. In this case, ADV and ADV_per_ models significantly reduced this vulnerability, which highlights the protective power of these adversarial methods in hiding such implicit information against invasive models that are specifically trained to infer this knowledge.

The same pattern was seen in the case of the other two demographic attributes, namely, gender and ethnicity. For ethnicity, the representation was less informative to the attacker network for the following two reasons:

1) A certain percentage of the patients had preferred not to state their ethnicity. Since the intention was to keep all the tasks binary, this category was treated as non-white which is clearly sub-optimal. This further complicates ethnicity prediction for the attacker.
2) There are limitations in the accuracy of documenting ethnicity by hospital staff during data collection, which may increase the amount of noise in the data.

However, even though the overall results are lower for the case of ethnicity, the ADV model still shows better privacy compared to the baseline. In such cases, the adversary is likely to rely on prior knowledge of the dataset or general information about the prevalence of ethnicity groups in the data, rather than the output of the encoder.

The adversarial setup came with only a minimal performance cost (Table IV) and proved robust both in the generalisability tests (Table X) and in external validation on highly imbalanced datasets (Section V-C2). More experiments (both at the level of data and model) are needed to ascertain whether the same general patterns can be seen under different conditions. Nonetheless, these methods are not tied to the specifics of the Base model and can be applied to any neural architecture. Furthermore, in the case of the ADV model, the protected attributes need not be demographic and theoretically any categorical feature of interest (or any feature that can be meaningfully quantised) can be used during training. Future work can also include experimenting with continuous features, in which the attacker would have to guess the features in a regression task.

In regards to the balance between subject privacy and the effectiveness of their diagnosis, any decisions would inevitably involve a trade-off. In this work, the boundaries of a leak are determined, in the sense that upper and lower bounds for the amount of possible leak the attacker can potentially exploit is explained. One cannot rely on absolute numbers only, as certain facts about the dataset might not be practical to hide (e.g. If an attacker knows the dataset was collected in Japan, it stands to reason that the demographic attribute of almost all patients would be ‘Asian’. Optimising for the model to completely hide this particular attribute is unnecessary and the knock to the performance would not be justified). Where the balance lies depends on several different factors including the following: The amount of background knowledge the attacker has of the model and dataset, the majority class stats for the individual protected attributes, how sensitive a particular piece of information is that would justify obscuring the representation to, as much as possible, protect it and accept the performance degradation, and last but not least, how much the performance is reduced after protecting an attribute. There is not one generic answer that can formulate all of the following into a single decision making formula and the specifics of each case determine where the balance should lie.

To conclude, in this paper two effective methods were introduced in order to protect sensitive attributes in a tabular dataset related to the task of predicting COVID-19 PCR test result based on routinely collected clinical data. The effectiveness of adversarial training was shown by assessing the proposed models against a comparable baseline both in the context of the main task where it showed performance scores that were by and large at the same level with the baselines and also in the context of privacy preservation where a trained attacker was employed to retrieve sensitive information by intercepting the content of the models' encoder. In the second scenario, the adversarially trained models consistently showed superior performance compared to the non-adversarial baseline.

For future work, this study can be expanded by including a larger set of feature ranges for each protected attribute and numerical features can also be included. Scenarios where the attacker has access to gradient updates or can fully observe all the weights from the entire network can also be explored. Future experiments can be designed with the aim for the attacker to reconstruct a larger set of attributes in a single run.
